# Development and validation of a newly developed nomogram for predicting the risk of deep vein thrombosis after surgery for lower limb fractures in elderly patients

**DOI:** 10.3389/fsurg.2023.1095505

**Published:** 2023-05-18

**Authors:** Shuai Han, Yunpeng Bai, Kun Jiao, Yongmin Qiu, Juhong Ding, Jun Zhang, Jingyun Hu, Haihan Song, Jiaqi Wang, Shufeng Li, Dapeng Feng, Jian Wang, Kai Li

**Affiliations:** ^1^ Department of Orthopedics, Shanghai Pudong New Area People's Hospital, Shanghai, China; ^2^ Department of Orthopedics, The Second Hospital of Dalian Medical University, Dalian, China; ^3^ Central Lab, Shanghai Key Laboratory of Pathogenic Fungi Medical Testing, Shanghai Pudong New Area People's Hospital, Shanghai, China; ^4^ Department of Orthopedic Surgery, Shandong Key Laboratory of Rheumatic Disease and Translational Medicine, The First Affiliated Hospital of Shandong First Medical University & Shandong Provincial Qianfoshan Hospital, Jinan, China

**Keywords:** nomograms, deep vein thrombosis, lower limb fractures, elderly patients, predictive factors

## Abstract

**Background:**

Prevention of deep vein thrombosis (DVT) is indispensable in the treatment of lower limb fractures during the perioperative period. This study aimed to develop and validate a novel model for predicting the risk of DVT in elderly patients after orthopedic surgeries for lower limb fractures.

**Methods:**

This observational study included 576 elderly patients with lower limb fractures who were surgically treated from January 2016 to December 2018. Eleven items affecting DVT were optimized by least absolute shrinkage and selection operator regression analysis. Multivariable logistic regression analysis was performed to construct a predictive model incorporating the selected features. C-index was applied to evaluate the discrimination. Decision curve analysis was employed to determine the clinical effectiveness of this model and calibration plot was applied to evaluate the calibration of this nomogram. The internal validation of this model was assessed by bootstrapping validation.

**Results:**

Predictive factors that affected the rate of DVT in this model included smoking, time from injury to surgery, operation time, blood transfusion, hip replacement arthroplasty, and D-dimer level after operation. The nomogram showed significant discrimination with a C-index of 0.919 (95% confidence interval: 0.893–0.946) and good calibration. Acceptable C-index value could still be reached in the interval validation. Decision curve analysis indicated that the DVT risk nomogram was useful within all possibility threshold.

**Conclusion:**

This newly developed nomogram could be used to predict the risk of DVT in elderly patients with lower limb fractures during the perioperative period.

## Introduction

Deep vein thrombosis (DVT) is a common complication in patients with lower limb fractures, which can result in fatal pulmonary embolism (PE) (1, 2). The incidence of DVT after surgery for lower limb fractures reported in previous studies ranges from 8.2 to 61.3% (3, 4). As most DVT cases are asymptomatic, early diagnosis is relatively difficult. If not prevented in time, some of the asymptomatic DVT may progress to symptomatic DVT with clinical symptoms such as redness, leg swelling, and tenderness. The consequence of symptomatic DVT often indicates a poor outcome and increases the risk of PE and mortality, and it is therefore highly important to prevent DVT in patients with lower limb fractures during the perioperative period.

DVT diagnosis has traditionally depended on digital subtraction angiograph (DSA). As a gold standard for diagnosis, DSA is a traumatic examination. Hence color Doppler ultrasonography is more frequently used in identifying DVT (5). Considering the actual situation, the objective and timely diagnosis of DVT is frequently delayed in daily practice (6, 7). Ageno et al. found that it required 5 days from symptom onset to the DVT diagnosis for 47.1% of patients and >10 days for 22.6% of patients (6). Hence, there is an urgent need to identify patient at high risk based on the related risk factors or admission laboratory biomarkers. By far, substantial efforts have been made to address this in orthopedics or other fields. The risk factors for DVT include older age, gender, obesity, smoking, cardiovascular disease, a cancer history, fracture location, delay in surgery and so on (8–10). Given so many associated risk factors, early accurate prediction of DVT by utilizing risk factors may be difficult to manage. Although previous studies have demonstrated that many risk factors are associated with DVT after lower limb fractures, they failed to translate the findings into accurate predictive model to identify DVT in elderly patients with lower limb fractures (7, 11–13). Indeed, these identified factors either relied too heavily on the subjective judgment of the assessors, or lacked well-validated biomarkers (14–16). Therefore, it is almost impossible to use these “isolated” risk factors to determine the “intuitive” and “real” possibility that one patient develops a DVT.

Caprini score has been frequently used to evaluate the risk of VTE in surgical patients. Although there is robust evidence to show its validity, it includes more than 30 risk factors in various aspects, which makes it too cumbersome and complex to allow for a rapid clinical diagnosis. Even worse, orthopedic surgeons were disappointed to find that elderly patients with lower limb fractures would always be classified as high-risk group (17–19). The purpose of this study was to develop a simple but valid predictive model to assess the risk of DVT in elderly patients with lower limb fractures, with the hope to provide a more accurate method for identifying and preventing DVT during the perioperative period.

## Patients and methods

The research protocol for this study was approved by the local ethics committee of Shanghai Pudong New Area People's Hospital. Patients were recruited from the Orthopedics Department from January 2016 to December 2018, and all the patients were diagnosed with new lower limb fractures. All participating patients signed informed consent.

The inclusion criteria were as follows: (1) age ≥60 years; (2) surgical treatment; and (3) internal implants. The exclusion criteria were as follows: (1) pathological fracture; (2) old fracture; (3) open fracture; (4) multiple complicated injuries; (5) anti-coagulation before injury; (6) hematological system disease; and (7) insufficient clinical or follow-up data.

After admission, all patients were treated with low molecular weight heparin (LMWH, 4,000 IU/d) for prophylaxis 12 h after injury. If the patients were hemodynamically unstable, LMWH heparin was not administered until the condition became stable. DVT screening of the lower extremities was performed using color Doppler ultrasonography before and after surgery.

The following data were collected: (1) demographics: gender, age, comorbidities, smoking history, and the time from injury to surgery; (2) location of the fracture: hip, knee, ankle and foot, femoral shaft, tibia and fibula shaft; (3) anesthesia method; (4) time of operation; (5) blood transfusion; (6) implant type; and (7) D-dimer level.

### Diagnostic methods and standards of DVT

Patients underwent venous color Doppler ultrasonography for DVT detection at 24 h after admission, 1 day before surgery, and 7 days after surgery. The deep veins of the proximal lower extremity were examined successively by using intermittent compression across the cross-section, from the femoral vein to the calf muscle venous plexus, and then successively along the longitudinal section of the above veins with the color doppler probe.

The diagnostic criteria of DVT (20): (1) the venous lumen could not be compressed; (2) the tone could be seen within the real echo; (3) the presence of blood flow signal filling defects in the lumen; (4) loss of phase in the blood flow spectrum; (5) incomplete obstruction of the vascular segment forming a “track” or “contour”; (6) no blood flow signal detectable in the vein segment or the presence of only a small amount of blood flow signal; and (7) pulse Doppler showing no blood flow or spectral changes with respiration.

### Statistical analysis

R version 4.2.1 (www.r-project.org) was used for statistical analysis. All data was presented as count (%). The least absolute shrinkage and selection operator (LASSO) regression, which is suitable for generating a relatively lower dimension model from high dimensional data, was used to select the potential prediction features in risk factors from patients with DVT in this study (21). In the LASSO model, features with nonzero coefficients were selected as significant predictors (22). Multivariable logistic regression analysis was utilized to build a predicting model. The potential features were evaluated by an odds ratio (OR), 95% confidence interval (CI) and corresponding *P*-values. Predictors with *P*-value < 0.05 were considered statistically significant and involved in the model (23). Calibration curves were used to assess the calibration of this nomogram. Harrell's C-index was applied to quantify the discrimination performance of this prediction nomogram. The relative C-index was corrected by bootstrapping validation (1,000 bootstrap resamples). Decision curve analysis was used to evaluate the clinical effectiveness of the DVT nomogram by comparing the net benefit under different threshold probabilities in the cohort of elderly patients with lower limb fracture (24). The net benefit was calculated by subtracting the proportion of false positive patients from the proportion of the true positive patients to identify who would really benefit from intervention by applying this nomogram.

## Results

### Patient characteristics

576 patients with lower limb fractures who received surgical treatment in our hospital from January 2016 to December 2018 were enrolled in this study, of whom 68 were diagnosed with DVT by color Doppler ultrasonography. The prevalence of DVT was 11.81%. All these 68 patients presented with asymptomatic DVT, and none of them developed PE. All the 576 patients were divided into a DVT group and a non-DVT group. All data of the patients including the demographics, disease and treatment features in the two groups are given in Table 1.

**Table 1:** Differences between demographic and clinical characteristics of DVT and non-DVT groups.

Clinical characteristics	non-DVT (*N* = 508)	DVT (*N* = 68)	*P*-value
**Sex**			
Female	329 (64.8%)	42 (61.8%)	0.726
Male	179 (35.2%)	26 (38.2%)	
**Age**			
60–70 years	225 (44.3%)	14 (20.6%)	<0.001^*^
71–80 years	159 (31.3%)	16 (23.5%)	
80 + years	124 (24.4%)	38 (55.9%)	
**Fracture site**			
Hip	457 (90.0%)	63 (92.6%)	0.078
Knee	29 (5.7%)	0 (0%)	
Ankle	22 (4.3%)	5 (7.4%)	
**Disease**			
None	224 (44.1%)	34 (50.0%)	0.743
Hypertension	15 (3.0%)	1 (1.5%)	
Diabetes	186 (36.6%)	22 (32.4%)	
Both	83 (16.3%)	11 (16.2%)	
**Smoke**			
No	441 (86.8%)	46 (67.6%)	<0.001^*^
Yes	67 (13.2%)	22 (32.4%)	
**Time from injury to surgery**			
0–6 days	425 (83.7%)	47 (69.1%)	0.014^*^
6–12 days	75 (14.8%)	19 (27.9%)	
>12 days	8 (1.6%)	2 (2.9%)	
**Anesthetization**			
Combined Spinal-Epidural	329 (64.8%)	42 (61.8%)	0.726
General	179 (35.2%)	26 (38.2%)	
**Surgery**			
Other	216 (42.5%)	3 (4.4%)	<0.001^*^
Intramedullary nail	124 (24.4%)	18 (26.5%)	
Hip replacement	168 (33.1%)	47 (69.1%)	
**Operation time**			
≤2 h	449 (88.4%)	40 (58.8%)	<0.001^*^
>2 h	59 (11.6%)	28 (41.2%)	
**Transfusion**			
No	329 (64.8%)	13 (19.1%)	<0.001^*^
Yes	179 (35.2%)	55 (80.9%)	
**D-dimer level**			
<0.5 ug	331 (65.2%)	18 (26.5%)	<0.001^*^
0.5–2.5 ug	35 (6.9%)	2 (2.9%)	
2.5–5 ug	59 (11.6%)	4 (5.9%)	
>5 ug	83 (16.3%)	44 (64.7%)	

* *P < 0.05.

### Feature selection

**Figure 1:**
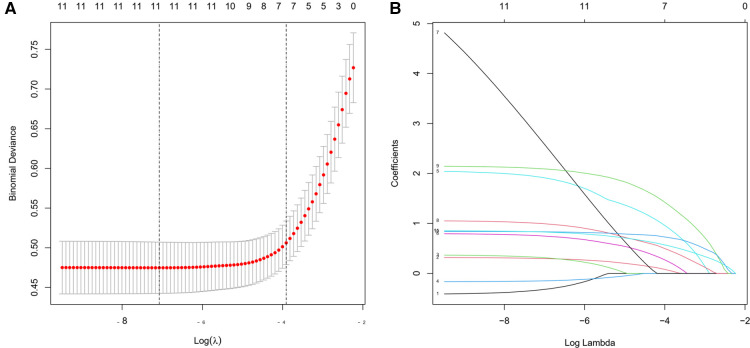
Demographic and clinical characteristic selection using the LASSO (least absolute shrinkage and selection operator) regression model.

11 features of the demographics, disease, and treatment features were reduced to 7 potential predictors on the basis of 576 patients in the cohort by LASSO regression model (Figures 1A,B). These features included age, smoking, time from injury to surgery, operation time, blood transfusion, implant type, and level of D-dimer.

### Development of an individualized prediction model

**Figure 2:**
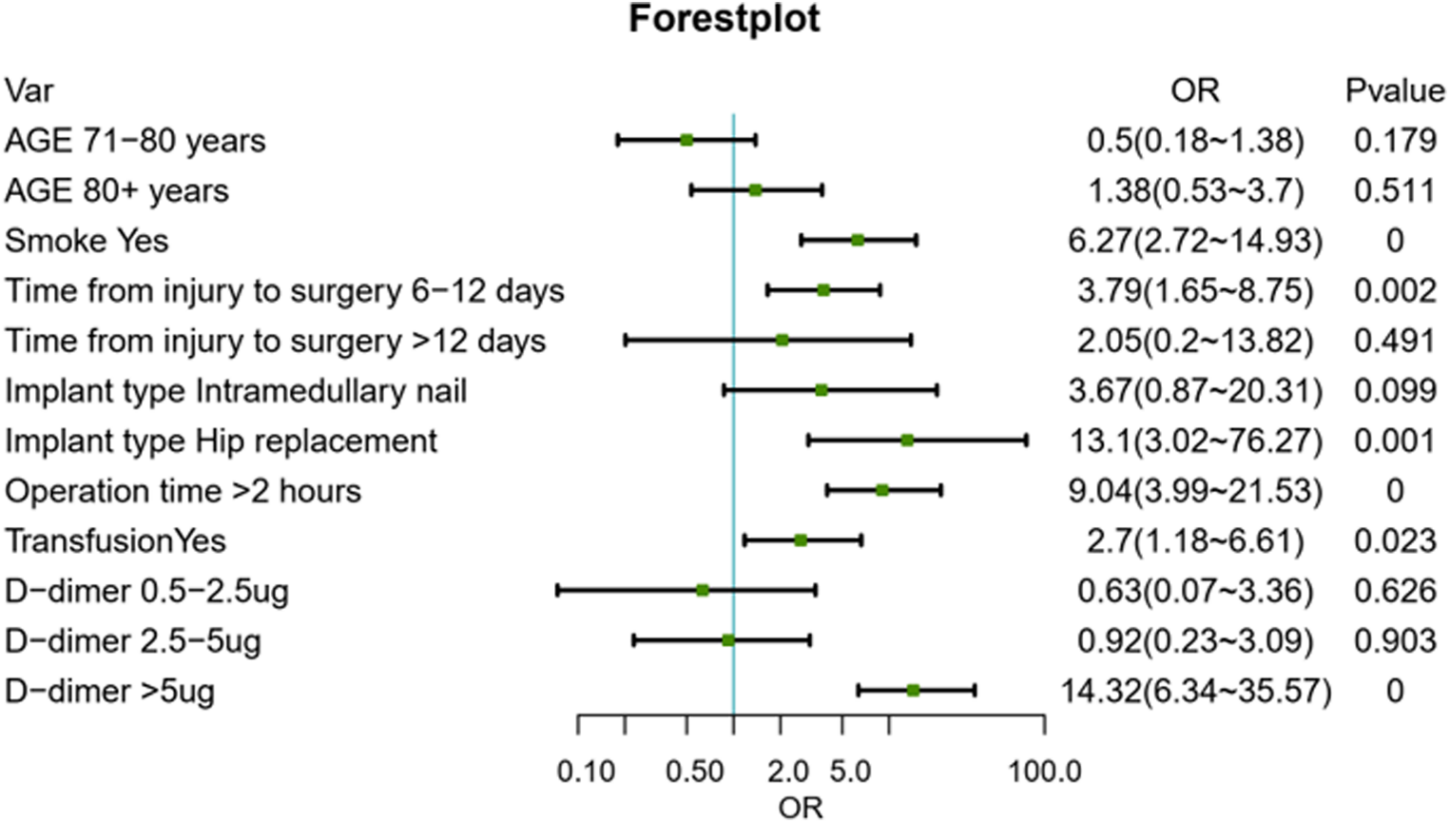
Forest plot showing the multivariable logistic regression. OR, odd ratio.

**Figure 3:**
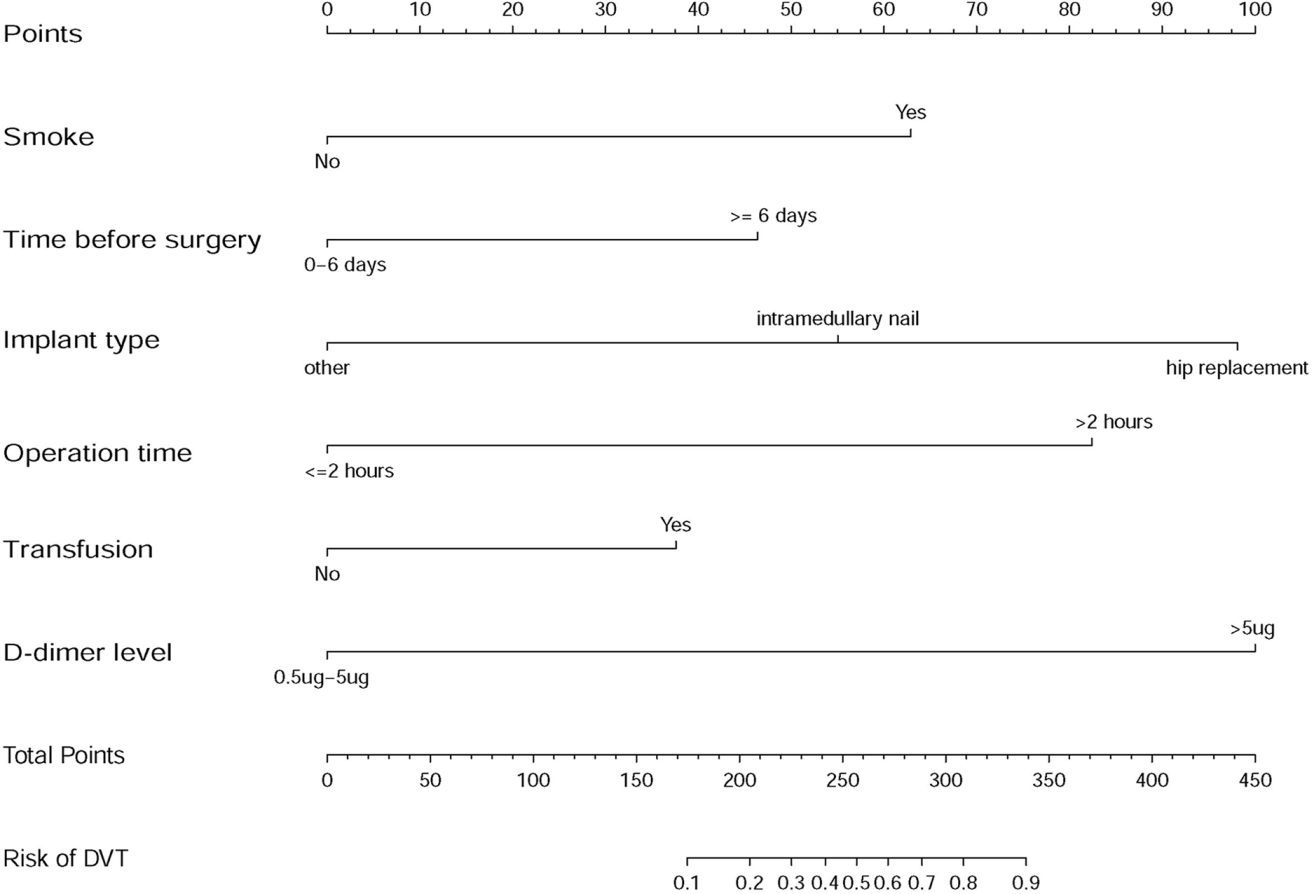
Developed DVT prediction nomogram. The sum of the scores of each predictor corresponds to the risk of DVT.

The results of the logistic regression analysis among age, smoking, time from injury to surgery, operation time, blood transfusion, implant type, and level of D-dimer are given in Figure 2. Based on the results of the forest plots, we removed age factor, and merged the non-significant sub group in the other factors. Then these adjusted predictors were incorporated into the nomogram as shown in Figure 3.

### Apparent performance of the DVT risk nomogram in the cohort

**Figure 4:**
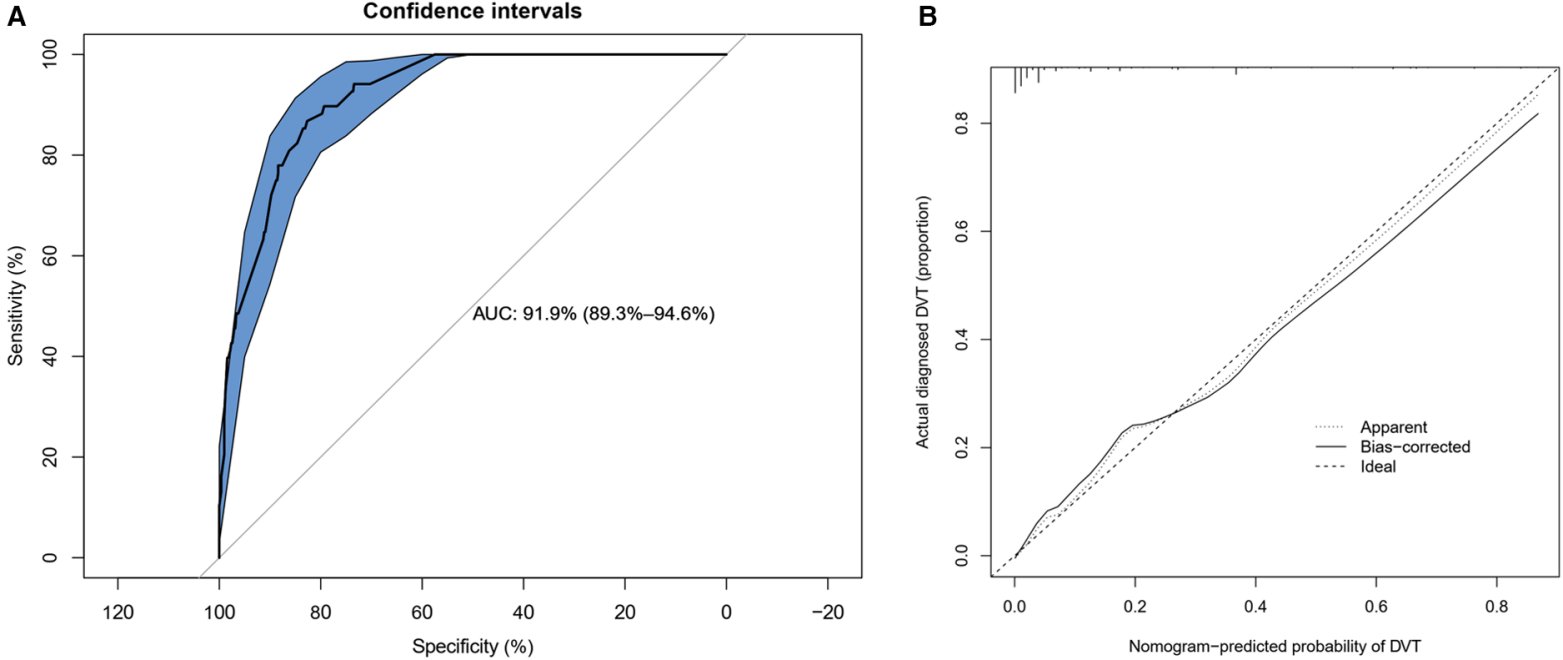
Calibration curves and ROC (receiver operating characteristic) curves of predicting DVT in the cohort. (**A**) ROC curves plotted to measure the discriminative capacity of nomogram. The area under curve (AUC) is positively correlated with the predictive accuracy of the nomogram. (**B**) Calibration curves of the DVT nomogram prediction in the cohort. In the calibration curve, the higher the overlap between the predicted curve and the ideal curve, the better the consistency between the predicted probability and the true probability.

The calibration curve of the DVT risk nomogram for the prediction of DVT risk in elderly patients with lower limb fractures demonstrated good agreement in this cohort (Figure 4). The C-index for the prediction nomogram was 0.919 (95%CI: 0.893–0.946) for the cohort, and was confirmed to be 0.9195 through bootstrapping validation, suggesting that the model had good discrimination. In the DVT risk nomogram, apparent performance manifested a good prediction capability.

### Clinical use

**Figure 5:**
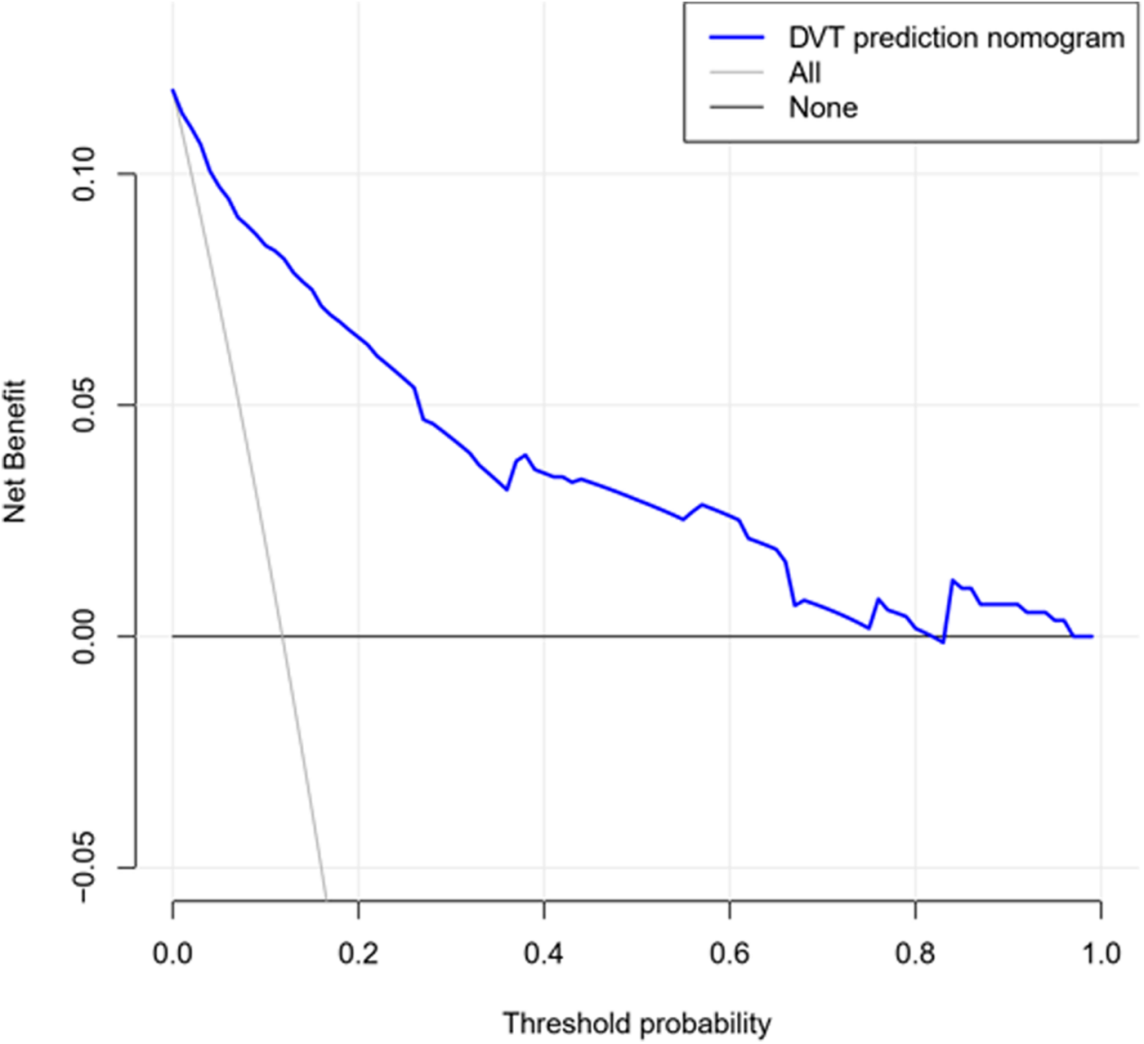
Decision curve used to measure the clinical usefulness. The X-axis represents the threshold probability and the Y-axis represents the net benefit. The blue line represents the DVT prediction nomogram. The grey line represents the assumption that DVT occurs in all patients. The black line represents the assumption that no patients suffer DVT. The range of threshold probabilities representing positive net benefit is obtained based on the corresponding points of the intersection of the blue line with the black line and grey line on the X-axis.

The decision curve analysis for the DVT nomogram is presented in Figure 5. The decision curve shows that within all threshold possibility, more benefits could be gained by using this nomogram to predict DVT, compared with the scheme.

## Discussion

A nomogram is a multi-index graphical calculating device and has been widely used as a prognostic device in cancer research and medicine, and it is a good tool to increase the accuracy of predicting prognosis and makes it more convenient to reach a clinical decision (25–27). To the best of our acknowledge, this study might be the first applied to DVT of lower limb fracture in elderly patients.

Lasso logistic regression is a useful alternative for variable reduction when collinearity is present. With this advantage, we developed and validated this predictive model for DVT risks in elderly patients with lower limb fractures by merely using these six easily available variables. Of all the collective factors, smoking, time from injury to surgery, operation time, blood transfusion, implant type, and D-dimer level were selected by multivariate logistic analysis to form the nomogram to facilitate the DVT individualized prediction of elderly patients with lower limb fractures. Good discrimination and high internal validation of this nomogram were assessed by the C-index. The high C-index value in the interval validation demonstrated that this nomogram might be useful and accurate during clinical practice. For such a high predictive performance of our model, overfitting must be a top concern (28–30). Regrettably, we were not able to find suitable publicly available datasets to verify this issue. The bootstrapping technique was thus used to perform internal validation and our model demonstrated quite stable performance, reflecting its ability to avoid overfitting (11, 31). Given the fact that there are only 6 predictors in our model and they are easy to implement, we are hoping that these results can be further validated by using clinical datasets from other investigators.

In the present study, 68 patients out of 576 were examined as asymptomatic proximal DVT by color Doppler ultrasonography above the popliteal vein, and the overall incidence of DVT was 11.81%. During the perioperative period of patients with lower limb fractures, many demographic, traumatic, and treatment features might be associated with the development of DVT. Our nomogram demonstrated that patients who had smoking history, delayed surgery, prolonged operation time, blood transfusion during operation, arthroplasty, and D-dimer level ≥5 µg/ml might be more likely to develop DVT (32).

As shown by previous studies (33, 34), smoking was associated with the occurrence of DVT, but whether it was an independent risk factor was also controversial (35, 36). Our study suggested that patients who had smoking habit in our series had a higher incidence of DVT, and the logistic regression analysis found smoking to be an independent predictor in this nomogram. Many previous studies indicated that delayed hip fracture operation on the elderly could significantly increase the incidence of DVT (2, 37–39). However, clinical practice shows that elderly patients often have other concomitant diseases, especially those over 80 years old, and eliminating the contraindications before operation often delays the operation. In our nomogram, a six-day or longer waiting before operation is an important factor contributing to the higher risk of DVT. Similar to the finding of previous studies (33, 40), longer operation time increased the risk of developing DVT, and more than two hours' operation time significantly increased the rate of DVT after lower limb fracture operation. In addition, intra-operative blood transfusion may also increase the risk of DVT, especially when the transfusion amount is more than 800 ml (41–44). The implant type also has impact on the risk of DVT. The implants used in this study included the plate and screw, intramedullary nail and artificial joint, of which the artificial joint was half hip or total hip replacement for femoral neck fracture. The incidence of DVT in hip replacement was higher than that in the internal fixation system, which was consistent with the previous reports (45) Previous studies demonstrated (46, 47) that the elevated level of D-dimer showed a significant association with DVT in patients with lower extremity fractures. In clinical practice, D-dimer is usually used as a reliable screening tool to predict DVT. But the precise level of D-dimer is still not clear (48, 49). It was found in our study that the rate of DVT significantly increased when the level of D-dimer exceeded 5 μg/ml.

The pathogenesis of DVT includes slow blood flow, a hypercoagulable state of blood and injury of the venous wall (50). The factors which can influent these pathological pathways may have impact on the risk of DVT. In traumatic orthopedics, fracture-associated peripheral capillary injury is often the initial factor of DVT formation. It is acknowledged that the incidence of DVT in lower limb fractures is much higher than that in other parts of the body, which is mainly related to the injury mechanism and the long-term bed confinement required for previous treatment (51). The incidence of DVT in elderly patients is significantly higher than that in the young patients due to their decreased motor function and cardiovascular function.

Several scoring systems have been built to help to predict the risk of DVT for general patients, such as Padua score and Caprini score (17–19). Unfortunately, elderly patients with lower limb fractures were all at the highest level according to above mentioned scoring systems (17–19). Based on this criterion alone, it is difficult for surgeons to make an effective and balanced decision and to know which patient should be focused on. That is why we not only made an incidence and risk factor analysis of DVT but also developed this nomogram of prediction model. For instance, the total points of a simple hip replacement patient were 98 and the incidence of DVT was much smaller than 10%, but if the patient had a smoking history and a high D-dimer level (>5 mg/ml), the total points would be 260(98 + 62 + 100) and the incidence of DVT would be almost 50%. Our model makes it possible to select patients who need early evaluation and intervention. We hope our work will provide an effective approach to improve accuracy and efficiency of predicting DVT so as to improve the prognosis of elderly patients with lower limb fracture.

There are several limitations in this study. First, our data were collected between January 2016 and December 2018, which might cause some selective bias. Second, we only collected the DVT consequence in the injury limbs and ignored the asymptomatic DVT in the uninjured leg. Third, we only performed color Doppler ultrasonography up to 7 days, and did not study long-term DVT results. Finally, although we have used bootstrap testing to examine internal validation of our nomogram, we are not sure whether this nomogram can be used universally in other hospitals or centers because of lack of external validation. Further studies are required to evaluate the long-term efficacy of this nomogram in larger-sample studies.

## Conclusion

This study has developed a relatively accurate nomogram to assess the risk of DVT in elderly patients with lower limb fractures. In clinical practice, surgeons can use this nomogram to estimate the risk of DVT of individual patients, and perform relevant prophylaxis to prevent the development of DVT. Nevertheless, external validation is needed to determine whether individual interventions based on this nomogram can reduce the DVT risk.

## Data Availability

The original contributions presented in the study are included in the article, further inquiries can be directed to the corresponding authors.

## References

[1] Alikhan R Peters F Wilmott R Cohen AT. Fatal pulmonary embolism in hospitalised patients: a necropsy review. J Clin Pathol. (2004) 57(12):1254–7. 10.1136/jcp.2003.013581 15563663 PMC1770519

[2] Decker S Weaver MJ. Deep venous thrombosis following different isolated lower extremity fractures: what is known about prevalences, locations, risk factors and prophylaxis. Eur J Trauma Emerg Surg. (2013) 39(6):591–8. 10.1007/s00068-013-0266-6 26815542

[3] Lee SY Ro du H Chung CY Lee KM Kwon SS Sung KH Incidence of deep vein thrombosis after major lower limb orthopedic surgery: analysis of a nationwide claim registry. Yonsei Med J. (2015) 56(1):139–45. 10.3349/ymj.2015.56.1.139 25510757 PMC4276747

[4] Zierler BK. Ultrasonography and diagnosis of venous thromboembolism. Circulation. (2004) 109(12 Suppl 1):I9–14. 10.1161/01.CIR.0000122870.22669.4a 15051663

[5] Needleman L Cronan JJ Lilly MP Merli GJ Adhikari S Hertzberg BS Ultrasound for lower extremity deep venous thrombosis: multidisciplinary recommendations from the society of radiologists in ultrasound consensus conference. Circulation. (2018) 137(14):1505–15. 10.1161/CIRCULATIONAHA.117.030687 29610129

[6] Ageno W Agnelli G Imberti D Moia M Palareti G Pistelli R Factors associated with the timing of diagnosis of venous thromboembolism: results from the MASTER registry. Thromb Res. (2008) 121(6):751–6. 10.1016/j.thromres.2007.08.009 17920107

[7] Elliott CG Goldhaber SZ Jensen RL. Delays in diagnosis of deep vein thrombosis and pulmonary embolism. Chest. (2005) 128(5):3372–6. 10.1378/chest.128.5.3372 16304286

[8] Anderson FA Jr Spencer FA. Risk factors for venous thromboembolism. Circulation. (2003) 107(23 Suppl 1):I9–16. 10.1161/01.CIR.0000078469.07362.E6 12814980

[9] Streiff MB Agnelli G Connors JM Crowther M Eichinger S Lopes R Guidance for the treatment of deep vein thrombosis and pulmonary embolism. J Thromb Thrombolysis. (2016) 41(1):32–67. 10.1007/s11239-015-1317-0 26780738 PMC4715858

[10] Heit JA Spencer FA White RH. The epidemiology of venous thromboembolism. J Thromb Thrombolysis. (2016) 41(1):3–14. 10.1007/s11239-015-1311-6 26780736 PMC4715842

[11] Hwang JP Lok AS Fisch MJ Cantor SB Barbo A Lin HY Models to predict hepatitis B virus infection among patients with cancer undergoing systemic anticancer therapy: a prospective cohort study. J Clin Oncol. (2018) 36(10):959–67. 10.1200/JCO.2017.75.6387 29447061 PMC7351320

[12] Xue Z Tu W Gao J Dong Z Yuan J Lang J. Optimal preoperative timing for prevention of deep vein thrombosis (DVT) in patients over 60 years of age with intertrochanteric fractures. Eur J Trauma Emerg Surg. (2022) 48(5):4197–203. 10.1007/s00068-022-01969-0 35445814

[13] Luo Z Chen W Li Y Wang X Zhang W Zhu Y Preoperative incidence and locations of deep venous thrombosis (DVT) of lower extremity following ankle fractures. Sci Rep. (2020) 10(1):10266. 10.1038/s41598-020-67365-z 32581237 PMC7314767

[14] Wang X Jiang Z Li Y Gao K Gao Y He X Prevalence of preoperative deep venous thrombosis (DVT) following elderly intertrochanteric fractures and development of a risk prediction model. BMC Musculoskelet Disord. (2022) 23(1):417. 10.1186/s12891-022-05381-y 35509097 PMC9065244

[15] Shekarchian S Notten P Barbati ME Van Laanen J Piao L Nieman F Development of a prediction model for deep vein thrombosis in a retrospective cohort of patients with suspected deep vein thrombosis in primary care. J Vasc Surg Venous Lymphat Disord. (2022) 10(5):1028–1036.e3. 10.1016/j.jvsv.2022.04.009 35644336

[16] Cheng X Lei X Wu H Luo H Fu X Gao Y Development and validation of a predictive nomogram for preoperative deep vein thrombosis (DVT) in isolated calcaneal fracture. Sci Rep. (2022) 12(1):5923. 10.1038/s41598-022-10002-8 35396396 PMC8993928

[17] Caprini JA. Identification of patient venous thromboembolism risk across the continuum of care. Clin Appl ThrombHemost. (2011) 17(6):590–9. 10.1177/1076029611404217 21593024

[18] Rogers FB Shackford SR Horst MA Miller JA Wu D Bradburn E Determining venous thromboembolic risk assessment for patients with trauma: the trauma embolic scoring system. J Trauma Acute Care Surg. (2012) 73(2):511–5. 10.1097/TA.0b013e3182588b54 23019680

[19] Tachino J Yamamoto K Shimizu K Shintani A Kimura A Ogura H Quick risk assessment profile (qRAP) is a prediction model for post-traumatic venous thromboembolism. Injury. (2019) 50(9):1540–4. 10.1016/j.injury.2019.06.020 31248583

[20] Hamper UM DeJong MR Scoutt LM. Ultrasound evaluation of the lower extremity veins. Radiol Clin North Am. (2007) 45(3):525–47, ix. 10.1016/j.rcl.2007.04.013 17601507

[21] Friedman J Hastie T Tibshirani R. Regularization paths for generalized linear models via coordinate descent. J Stat Softw. (2010) 33(1):1–22. 10.18637/jss.v033.i01 20808728 PMC2929880

[22] Kidd AC McGettrick M Tsim S Halligan DL Bylesjo M Blyth KG. Survival prediction in mesothelioma using a scalable lasso regression model: instructions for use and initial performance using clinical predictors. BMJ Open Respir Res. (2018) 5(1):e000240. 10.1200/JCO.2007.12.9791 29468073 PMC5812388

[23] Iasonos A Schrag D Raj GV Panageas KS. How to build and interpret a nomogram for cancer prognosis. J Clin Oncol. (2008) 26(8):1364–70. 10.1200/JCO.2007.12.9791 18323559

[24] Mo S Dai W Xiang W Li Q Wang R Cai G. Predictive factors of synchronous colorectal peritoneal metastases: development of a nomogram and study of its utilities using decision curve analysis. Int J Surg. (2018) 54(Pt A):149–55. 10.1016/j.ijsu.2018.04.051 29730071

[25] Park H Lim Y Ko ES Cho HH Lee JE Han BK Radiomics signature on magnetic resonance imaging: association with disease-free survival in patients with invasive breast cancer. Clin Cancer Res. (2018) 24(19):4705–14. 10.1158/1078-0432.CCR-17-3783 29914892

[26] Huang JL Fu YP Jing CY Yi Y Sun J Gan W A novel and validated prognostic nomogram based on liver fibrosis and tumor burden for patients with hepatocellular carcinoma after curative resection. J Surg Oncol. (2018) 117(4):625–33. 10.1002/jso.24895 29165812

[27] García-Tello A Gimbernat H Redondo C Meilán E Arana DM Cacho J Prediction of infection caused by extended-spectrum beta-lactamase-producing Enterobacteriaceae: development of a clinical decision-making nomogram. Scand J Urol. (2018) 52(1):70–5. 10.1080/21681805.2017.1373698 28893132

[28] Segar MW Jaeger BC Patel KV Nambi V Ndumele CE Correa A Development and validation of machine learning-based race-specific models to predict 10-year risk of heart failure: a multicohort analysis. Circulation. (2021) 143(24):2370–83. 10.1161/CIRCULATIONAHA.120.053134 33845593 PMC9976274

[29] Ramírez-Aportela E Maluenda D Fonseca YC Conesa P Marabini R Heymann JB FSC-Q: a CryoEM map-to-atomic model quality validation based on the local Fourier shell correlation. Nat Commun. (2021) 12(1):42. 10.1038/s41467-020-20295-w 33397925 PMC7782520

[30] Biffi A Rattani A Anderson CD Ayres AM Gurol EM Greenberg SM Delayed seizures after intracerebral haemorrhage. Brain. (2016) 139(Pt 10):2694–705. 10.1093/brain/aww199 27497491 PMC5035821

[31] Steyerberg EW Harrell FE Jr Borsboom GJ Eijkemans MJ Vergouwe Y Habbema JD. Internal validation of predictive models: efficiency of some procedures for logistic regression analysis. J Clin Epidemiol. (2001) 54(8):774–81. 10.1016/S0895-4356(01)00341-9 11470385

[32] Chan NC Stehouwer AC Hirsh J Ginsberg JS Alazzoni A Coppens M Lack of consistency in the relationship between asymptomatic DVT detected by venography and symptomatic VTE in thromboprophylaxis trials. ThrombHaemost. (2015) 114(5):1049–57. 10.1160/TH14-12-1006 26134342

[33] Li Q Chen X Wang Y Li L. Analysis of the occurrence of deep venous thrombosis in lower extremity fractures: a clinical study. Pak J Med Sci. (2018) 34(4):828–32. 10.12669/pjms.344.14752 30190736 PMC6115566

[34] Syed FF Beeching NJ. Lower-limb deep-vein thrombosis in a general hospital: risk factors, outcomes and the contribution of intravenous drug use. QJM. (2005) 98(2):139–45. 10.1093/qjmed/hci020 15655094

[35] Whiting PS White-Dzuro GA Greenberg SE VanHouten JP Avilucea FR Obremskey WT Risk factors for deep venous thrombosis following orthopaedic trauma surgery: an analysis of 56,000 patients. Arch Trauma Res. (2016) 5(1):e32915. 10.5812/atr.32915 27148502 PMC4853595

[36] Khazi ZM An Q Duchman KR Westermann RW. Incidence and risk factors for venous thromboembolism following hip arthroscopy: a population-based study. Arthroscopy. (2019) 35(8):2380–2384.e1. 10.1016/j.arthro.2019.03.054 31395174

[37] Zhang W Huai Y Wang W Xue K Chen L Chen C A retrospective cohort study on the risk factors of deep vein thrombosis (DVT) for patients with traumatic fracture at honghui hospital. BMJ Open. (2019) 9(3):e024247. 10.1136/bmjopen-2018-024247 30833318 PMC6443064

[38] Hill J Treasure T. Reducing the risk of venous thromboembolism (deep vein thrombosis and pulmonary embolism) in patients admitted to hospital: summary of the NICE guideline. Heart. (2010) 96(11):879–82. 10.1136/hrt.2010.198275 20478866

[39] Hefley FG Jr Nelson CL Puskarich-May CL. Effect of delayed admission to the hospital on the preoperative prevalence of deep-vein thrombosis associated with fractures about the hip. J Bone Joint Surg Am. (1996) 78(4):581–3. 10.2106/00004623-199604000-00012 8609137

[40] Tateiwa T Ishida T Masaoka T Shishido T Takahashi Y Onozuka A Clinical course of asymptomatic deep vein thrombosis after total knee arthroplasty in Japanese patients. J Orthop Surg. (2019) 27(2):2309499019848095. 10.1177/2309499019848095 31084257

[41] Ghazi L Schwann TA Engoren MC Habib RH. Role of blood transfusion product type and amount in deep vein thrombosis after cardiac surgery. Thromb Res. (2015) 136(6):1204–10. 10.1016/j.thromres.2015.10.041 26553018

[42] Jiang T Song K Yao Y Pan P Jiang Q. Perioperative allogenic blood transfusion increases the incidence of postoperative deep vein thrombosis in total knee and hip arthroplasty. J Orthop Surg Res. (2019) 14(1):235. 10.1186/s13018-019-1270-2 31337430 PMC6651957

[43] Lin SY Chang YL Yeh HC Lin CL Kao CH. Blood transfusion and risk of venous thromboembolism: a population-based cohort study. Thromb Haemost. (2020) 120(1):156–67. 10.1055/s-0039-1697664 31639832

[44] Goel R Patel EU Cushing MM Frank SM Ness PM Takemoto CM Association of perioperative red blood cell transfusions with venous thromboembolism in a north American registry. JAMA Surg. (2018) 153(9):826–33. 10.1001/jamasurg.2018.1565 29898202 PMC6233649

[45] Kim JS. Deep vein thrombosis prophylaxis after total hip arthroplasty in Asian patients. Hip Pelvis. (2018) 30(4):197–201. 10.5371/hp.2018.30.4.197 30534537 PMC6284075

[46] Singer AJ Zheng H Francis S Fermann GJ Chang AM Parry BA D-dimer levels in VTE patients with distal and proximal clots. Am J Emerg Med. (2019) 37(1):33–7. 10.1016/j.ajem.2018.04.040 29703562

[47] Jiang Y Li J Liu Y Li YC Zhang WG. Risk factors for deep vein thrombosis after orthopedic surgery and the diagnostic value of D-dimer. Ann Vasc Surg. (2015) 29(4):675–81. 10.1016/j.avsg.2014.12.022 25728333

[48] Nata S Hiromatsu S Shintani Y Ohno T Akashi H Tanaka H. D-dimer value more than 3.6 μg/ml is highly possible existence deep vein thrombosis. Kurume Med J. (2013) 60(2):47–51. 10.2739/kurumemedj.MS61009 24464132

[49] Yoshiiwa T Miyazaki M Takita C Itonaga I Tsumura H. Analysis of measured D-dimer levels for detection of deep venous thrombosis and pulmonary embolism after spinal surgery. J Spinal Disord Tech. (2011) 24(4):E35–9. 10.1097/BSD.0b013e3181f60603 20975598

[50] Aviram M Viener A Brook JG. Reduced plasma high-density lipoprotein and increased platelet activity in arterial versus venous blood. Postgrad Med J. (1987) 63(736):91–4. 10.1136/pgmj.63.736.91 3671249 PMC2428243

[51] Shin WC Woo SH Lee SJ Lee JS Kim C Suh KT. Preoperative prevalence of and risk factors for venous thromboembolism in patients with a hip fracture: an indirect multidetector CT venography study. J Bone Joint Surg Am. (2016) 98(24):2089–95. 10.2106/JBJS.15.01329 28002372

